# Meta-analytic evaluation of complications and longevity of bone-anchored prostheses in lower extremity amputees: does design matter?

**DOI:** 10.1007/s12306-026-00946-9

**Published:** 2026-01-14

**Authors:** Hugh Milchem, Yasmine J. Khair, Maamoun Adra, Shayndhan S. Sivanathan, Shreehari Suresh, Dilip K. Vankayalapati, Aslam Mohamed Haroon, Christian A. Than, Nadim Tarazi, Hayato Nakanishi, Peter J. Smitham

**Affiliations:** 1 Medical School, University of Nicosia, Nicosia, 2408 Cyprus; 2 Oxford University Hospitals NHS Foundation Trust, John Radcliffe Hospital, Oxford, UK; 3 School of Biomedical Sciences, The University of Queensland, St Lucia, 4072 Brisbane Australia; 4 Cambridge University Hospitals NHS Foundation Trust, Addenbrookes Hospital, Cambridge, CB2 0QQ UK; 5 Department of Surgery, Mayo Clinic, Rochester, USA; 6 Orthopaedic and Trauma Department, Royal Adelaide Hospital, Adelaide, 5000 Australia; 7 Discipline of Orthopaedics, University of Adelaide, Adelaide, 5000 Australia; 8 Hull University Teaching Hospital NHS Trust, Hull Royal Infirmary, Anlaby Road, Hull, East Yorkshire HU3 2JZ UK

**Keywords:** Osseointegration, Bone-anchored prosthesis, Amputation, Prosthesis, Implant

## Abstract

Bone-anchored prostheses (BAPs) are an alternative option for lower-limb amputees with problematic suspended socket prostheses (SSPs). We sought to meta-analytically quantify complication burden and revision-free survival for BAPs, whilst investigating possible differences in complication rates between common screw-fit (OPRA) and press-fit (ILP/OPL) designs. A multi-database search of PubMed, EMBASE, CiNAHL, Cochrane Cochrane Central Register of Controlled Trials, Cochrane Database of Systematic Reviews, Scopus and Web of Science from inception to September 2025 was conducted. Proportions were pooled using a random-effects model. This review was registered in PROPSPERO (ID: CRD42024507070). A total of 22 studies reporting 979 patients were included. An overall complication rate of 65% (95% CI: 0.53–0.75, I^2^ = 69%) was found. The most common complication was breakage of the abutment or dual-cone adaptor (DCA) (0.38, 95% CI: 0.19–0.62, I^2^ = 97%), which constituted 94% of all mechanical failures (95% CI: 0.87–0.98, I^2^ = 51%). Second was infection (0.36, 95% CI: 0.25–0.50, I^2^ = 92%) with 72% of infections being grade I (95% CI: 0.57–0.83, I^2^ = 72%). Estimated revision-free survival was 89% (95% CI: 0.84–0.93, I^2^ = 0%) at five years and 77% (95% CI: 0.70–0.82, I^2^ = 0%) at ten years. The largest difference between implant designs was for overall proportion of mechanical failure, OPRA at 68% (95% CI: 0.34–0.90, I^2^ = 98%), ILP/OPL at 19% (0.09–0.36, I^2^ = 85%). BAPs do carry appreciable risks for superficial infection and mechanical (abutment/DCA) breakage, with screw-fit designs appearing to have a higher mechanical failure rate. However, the revision-free survival rate overall remains acceptable up to 10 years demonstrating the viability of BAPs in lower-limb amputees.

**Supplementary Information:**

The online version contains supplementary material available at 10.1007/s12306-026-00946-9.

## Introduction

Lower extremity amputation is a sequela of both traumatic and non-traumatic pathologies. In 2019 alone, over 1 million patients worldwide underwent traumatic amputation of at least one lower limb [1]. Restoration of functionality for amputees, post-traumatic or otherwise, has traditionally been achieved through use of suspended socket prostheses (SSPs) [2]. They continue to be the primary management option but suffer inherent limitations, including discomfort, local skin pain and pressure sores or ulceration [3]. Many subsequently avoid prosthetic use, at the expense of mobility and quality of life [4]. It is estimated up to half of lower-limb amputees do not regularly employ their prosthesis [5]. These challenges have prompted clinicians and patients alike to seek alternatives.

In 1990, Rickard Brånemark attempted to implant two bone-anchored prostheses (BAPs) in a woman with traumatic bilateral transfemoral amputation. The procedure was successful, and the first demonstrating long-term viability [6]. The terms “bone-anchored”, “osseointegrated” and “transcutaneous” prostheses are now commonplace and essentially identical. Since 1990, offerings have expanded to several different implant systems administered by multiple centres worldwide. Some centres now boast patient cohorts with robust follow-up data spanning more than a decade [7], highlighting both benefits and limitations of this novel approach.

BAPs successfully mitigate several problems associated with SSPs. Physiological weight distribution is made possible, and range of motion and osseoperception are appreciably improved. This is reflected in improved quality of life and stable prosthetic control [8–11]. However, infection and implant breakage have been identified as complications. Clinically, BAPs are usually offered to patients experiencing persistent problems with SSPs and are still a relatively new and evolving technology. As such, research demands regular evaluation to quantify previously identified benefits and limitations. To the best of the authors’ knowledge, no study has comprehensively meta-analysed survival and complication rates of BAPs in lower-limb amputees. Accordingly, the findings here aim to contribute to a growing literature base aimed at guiding clinical decision making and expectations regarding the use of BAPs.

## Methods

### Search strategy and data sources

In accordance with Preferred Reporting Items for Systematic Reviews and Meta-analyses (PRISMA) guidelines, a comprehensive search of several databases from their inception to 1 September 2025 was performed [12]. Databases included PubMed, EMBASE, CiNAHL, Cochrane Central Register of Controlled Trials, Cochrane Database of Systematic Reviews, Scopus and Web of Science. An experienced medical reference librarian designed and conducted the search with input from the study's principal investigator. Controlled vocabulary supplemented with keywords was used to search for studies describing outcomes of lower-limb amputees managed with implantation of a bone-anchored prosthesis. No language restriction was placed on the search, and non-English articles were machine-translated. The actual strategy listing all search terms used and their combinations is provided in Supplementary Information: Item 1. This review was registered prospectively with PROSPERO (CRD42024507070).

### Eligibility criteria and quality assessment

Eligible studies were required to meet the following inclusion criteria: (1) Patients > 18 years old, with transfemoral or transtibial amputation (TFA or TTA) managed with implantation of any type of bone-anchored prosthesis. Knee disarticulations were also considered acceptable. (2) Report on at least one primary outcome: adverse events/complications or survival. (3) Employ a randomised, comparative, retrospective, prospective or case-series study design. Case series with more than five patients were included. Case reports, abstracts, conference abstracts and studies with overlapping patient data were excluded. Studies including upper-limb amputees were excluded. Studies were screened for inclusion and extracted independently by two separate authors (HM, YJK, MA, SSS, AMH, SS). Assessment for study quality and bias was undertaken in the domains confounding, participant selection, missing data, measurement of outcomes and selection of reported results. Each study was quality-evaluated using the risk of bias in non-randomised studies of interventions (ROBINS-I) tool by two separate authors (YJK, MA, SSS, AMH, SS) [13]. Any disagreement on the quality of a study was resolved through discussion with a third author (HM). The results of quality assessment of all included studies and methodology used are available in Supplementary Information: Fig. S1. Publication bias was assessed for all analyses including ten or more studies through generation of contour-enhanced funnel plots (Supplementary Information: Fig. S2).

### Outcomes

Adverse events/complications and implant survival were the outcome variables of interest. Adverse events were extracted as infectious and non-infectious (including mechanical). Infection data were extracted from all papers, including papers utilising the four-stage OGAAP (Osseointegration Group of Australia Accelerated Protocol) infection scale designed by Al Muderis et al. [14]. Papers with adequate uncategorised infection data were extracted into the OGAAP scale. Stage I describes superficial soft tissue infection, stage II deeper tissue infection, stage III bone infection, and stage IV septic loosening of the intramedullary stem (implant failure). Studies [15–20] that only reported partial infection data, i.e. only superficial or only deep infections were excluded from the analysis.

Non-infectious complications included breakage of the intramedullary implant stem or abutment/dual-cone adaptor (DCA) (considered mechanical), bone fracture (periprosthetic or other), stoma/stump revision, aseptic implant loosening, tissue hypergranulation and implant-related (i.e. not phantom) pain.

Implant failure was defined as any event necessitating explantation (revision or permanent removal) of the implant after initial surgeries. Estimated implant survival was measured by extracting time-to-event data reconstructed from studies with Kaplan–Meier curves. If survival data were reported separately before and after development of a formal protocol, post-protocol data were used.

### Data extraction

Adverse events and implant survival were extracted at short-, mid- and long-term follow-up; defined as 12 months, 13–59 months and ≥ 60 months, respectively. Survival data were extracted across all follow-up periods and separately at 1-, 3-, 5- and 10-years. Patient data were reconstructed and digitised from any papers that reported survival in a Kaplan–Meier curve using WebPlotDigitizer version 4 (https://automeris.io/WebPlotDigitizer/) and computed using the algorithm by Guyot and colleagues [21]. Two different authors independently checked all extracted data (HM, YJK, MA, SSS, AMH, SS).

### Statistical analysis

Proportions of data were pooled using the generic inverse variance method of DerSimonian and Laird. Proportions underwent logit transformation prior to meta-analysis. Between-study variance *τ*^2^ was calculated using the restricted maximum likelihood (REML) method. A value of I^2^ of 0–25% indicates minimal heterogeneity, 26–50% moderate heterogeneity and 51–100% substantial heterogeneity. The random-effects model was used. Subgroup analysis was performed using the same methodology. Leave-one-out sensitivity analysis was performed, screening for any studies with an exaggerated effect size. If mean and standard deviation (SD) were unavailable, median was converted to mean using formulas from the Cochrane Handbook for Systematic Reviews of Interventions. If SD was not available or extractable, the reported mean was omitted from the calculation. Authors were contacted three times to obtain required information omitted in published articles. Subgroup analysis using a random-effects model was undertaken with intent to investigate differences in outcomes between major screw-fit (OPRA) and press-fit (ILP/OPL) implant designs. Data analysis, forest plot and funnel plot generation were performed in the RStudio® 2024.04.2 environment (Posit Software) for R 4.4.0 (R Core Team, 2024, Vienna, Austria), using the “meta” package (Balduzzi S, Rücker G, Schwarzer G, 2019).

## Results

### Study selection and characteristics

**Fig. 1:**
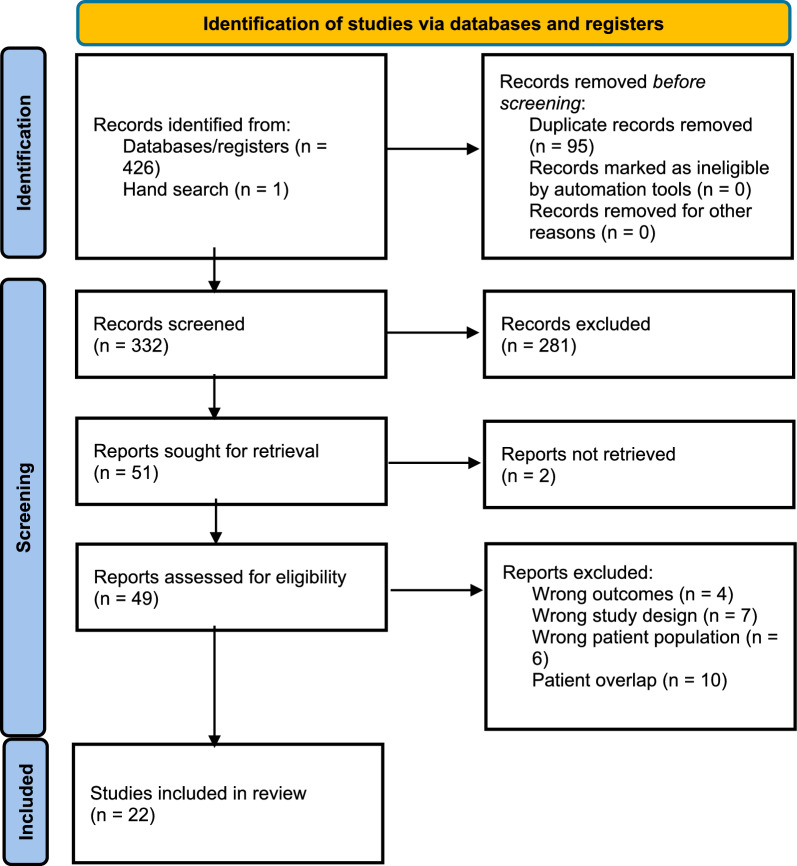
PRISMA chart

The initial literature search returned 331 articles, 22 of which reported on 979 patients comprising 71% male (n = 699), 27% female (n = 266) and 2% (n = 14) not reported. Each stage of the selection process is detailed in the PRISMA flow chart (Fig. 1). Most included articles were retrospective cohort studies (n = 13) [17, 18, 22–32], followed by prospective cohort studies (n = 8) [14–16, 33–37]. One retrospective case-series [38] was also included. Baseline characteristics of all included studies are summarised in Table 1. All studies only reported on patients with a BAP implanted in the lower extremity. Included studies with overlap had the overlapping portion of their cohort deducted [14, 15, 17, 23, 33, 34]. Studies indicating near-total patient overlap or unclear overlap were excluded outright after initial inclusion.

**Table 1:** Study characteristics

Study	Country	Patients (n)	Males/Females (n)	Mean age ± SD (years)	Transfemoral amputees (n)	Transtibial amputees (n)	Implant type	Surgical protocol	Follow-up (yrs)	Study design
Akhtar et al., 2022	UK	10	3:2	62.4 ± 11.4	10	0	ILP/OPL	Single-stage	5.2 ± 2.5	Retrospective
Aschoff et al., 2016	Germany	86	34:9	NR	79	7	Endo-Exo/ILP	NR	NR	Retrospective
Brånemark et al., 2019*	Sweden	51	28:23	44.0 ± 12.0	51	0	OPRA	Two-stage	5	Prospective
Corona et al., 2024	USA	11	7:4	53 ± 11.75	11	0	OPL (A)	Single-stage	2	Prospective
Hagberg et al.,2020*	Sweden	111	26:11	44.6 ± 12.6	111	0	OPRA	Two-stage	15	Prospective
Hagberg et al.,2022*	Sweden	51	28:23	44.0 ± 12.0	51	0	OPRA	Two-stage	10	Prospective
Hansen et al., 2019	Denmark	17	11:6	50.0 ± 8.5	17	0	OPRA	Two-stage	NR	Prospective
Juhnke & Aschoff 2015†	Germany	74	59:15	45.5 ± 14.7	67	7	Endo-Exo/ILP	NR	NR	Retrospective
Juhnke, Beck et al., 2015†	Germany	69	56:13	45.4 ± 12.3	69	0	ILP	Two-stage	NR	Retrospective
Matthews et al., 2019	UK	18	5:1	34.8 ± 9.3	18	0	OPRA	Two-stage	NR	Retrospective
Marano et al., 2020	USA	14	NR	50.0 ± 11.0	12	2	NR	Single-stage	NR	Retrospective
McMenemy et al., 2020	UK	7	7:0	28.0 ± 6.7	7	0	OPL	Single-stage	≥ 2	Retrospective
Mohamed et al., 2022	Netherlands	58	41:17	51.0 ± 13.0	53	0	ILP	Two-stage	5	Retrospective
Muderis et al., 2016‡	Australia	44	35:9	47.0 ± 13.0	44	0	ILP	Two-stage	≥ 2	Prospective
Potter et al., 2025	USA	37	34:3	38 ± 10	37	0	OPRA	Two-stage	≥ 2	Prospective
Reetz et al., 2020	Netherlands	39	10:3	48.7 ± 13.9	39	0	ILP	Two-stage	5	Retrospective
Reif et al., 2021	USA	31	19:12	50.3 ± 12.9	18	13	OPL	Two-stage	0.5	Retrospective
Rojas et al., 2021	Chile	21	6:1	43.0 ± 8.7	21	0	OPRA	Two-stage	≥ 5	Retrospective
Sinclair et al., 2022	USA	10	10:0	48.8 ± 12.0	10	0	POP	Two-stage	NR	Prospective
Sreedharan et al., 2021	Australia	18	2:1	NR	18	0	OPRA	Two-stage	NR	Retrospective
Thouvenin et al., 2024	France	17	7:10	41.0 ± 9.3	14	3	OPRA	Two-stage	15	Retrospective
van Vliet-Bockting et al., 2025	Netherlands	206	74:29	54 ± 13	139**	70**	ILP/OPL	Mixed	≥ 2	Retrospective

^*^Overlapping patient population. †Overlapping patient population. ‡This article also reports an overlapping cohort from the Netherlands which was excluded. **Number of limbs

ILP: Integral-leg-prosthesis (Orthodynamics), OPL: Osseointegration prosthetic limb system (Permedica), OTI: Osseointegration tibia implant, OPRA: Osseointegrated prostheses for the rehabilitation of amputees (Integrum), POP: Percutaneous osseointegrated prosthesis (DJO Surgical), NR: Not reported, SD: standard deviation

Nine studies (n = 369) [14–16, 18, 27, 28, 30, 34, 35] reported a mean time of 10.6 years (95% CI: 8.9–12.7, I^2^ = 81%) between amputation and first BAP surgery. The average age across 16 non-overlapping studies was 46.2 years (95% CI: 42.3–50.4, I^2^ = 92%). A total of eleven studies utilised Integral-Leg-Prosthesis (ILP)/Osseointegrated Prosthetic Limb (OPL) [14, 17, 18, 22, 23, 26–28, 32, 36, 38]. A total of nine studies utilised Osseointegrated Prostheses for the Rehabilitation of Amputees (OPRA) [15, 16, 25, 29–31, 33, 34, 37]. Two studies utilised other variants of protheses [24, 35].

### Risk of bias

Results of ROBINS-I quality assessment for included studies are provided in Supplementary Information (SI): Fig. S1. Three studies were judged to be at low risk of bias [16, 29, 34], 10 moderate [14, 15, 25–28, 30, 31, 33, 35] and 9 at serious risk of bias [17, 18, 22–24, 32, 36–38]. Any determined to have critical risk of bias were ineligible for inclusion in the meta-analysis. Serious bias was identified due to confounding [18, 22, 23, 32, 36, 38] and selection of participants [17, 24].

### Overall complications/adverse events

An overall complication rate of 65% (95% CI: 0.53–0.75, I^2^ = 69%) was found from 10 included studies [15, 16, 18, 24, 27, 28, 32, 35, 36, 38].

Across all follow-up periods, breakage of only the abutment/DCA constituted the most frequent adverse event, and highest frequency non-infectious mechanical complication at 38% (n = 598) (95% CI: 0.19–0.62, I^2^ = 97%)**.** Infections of any type were the second highest adverse event, at 36% (n = 530) (95% CI: 0.25–0.50, I^2^ = 92%). Overall mechanical failure (abutment/DCA or implant breakage) was next greatest at 37% (n = 607) (95% CI: 0.19–0.60, I^2^ = 96%). Stump revision followed as the most frequent non-mechanical, non-infectious complication at 17% (n = 98) (95% CI: 0.08–0.32, I^2^ = 87%). Wound hypergranulation represented 12% (n = 56) (95% CI: 0.05–0.24, I^2^ = 86%), whilst BAP-related pain a lesser proportion at 6% (n = 24) (95% CI: 0.02–0.22, I^2^ = 86%). Bone fracture constituted a small proportion of 4% (n = 33) (95% CI: 0.02–0.06, I^2^ = 35%). Aseptic implant loosening was uncommon at 3% (n = 15) (95% CI 0.01–0.07, I^2^ = 58%). Implant breakage alone was the least common complication at 1% (n = 5) (95% CI 0.01–0.03, I^2^ = 17%). The breakdown of complications is summarised in Table 2.

**Table 2:** Complications, any follow-up

Complication	Proportion	95% CI	Events	I^2^	Included studies (n)	Sample size (n)
Overall	0.65	0.53–0.75	308	69%	10	456
Any infection	0.36	0.25–0.50	530	92%	14	1173
Abutment/dual-cone breakage	0.38	0.19–0.62	598	97%	12	1104
Any mechanical failure	0.37	0.19–0.60	607	96%	13	1114
Stump revision	0.17	0.08–0.32	98	87%	10	629
Hypergranulation**	0.12	0.05–0.24	56	86%	3	526
Pain	0.06	0.02–0.22	24	86%	6	496
Bone fracture	0.04	0.02–0.06	33	35%	13	1139
Aseptic loosening	0.03	0.01–0.07	15	58%	10	1040
Implant breakage*	0.01	0.01–0.03	5	17%	10	892
*Subgroup ILP/OPL implants*						
Overall	0.71	0.56–0.83	225	58%	6	304
Any infection	0.51	0.38–0.63	369	90%	6	690
Stump revision	0.26	0.11–0.47	83	87%	7	420
Any mechanical failure	0.19	0.09–0.36	49	85%	5	340
Dual-cone breakage	0.18	0.08–0.35	46	85%	5	340
Bone fracture	0.05	0.03–0.09	21	0%	7	448
Pain	0.02	0.01–0.05	4	21%	2	284
Aseptic loosening	0.02	0.00–0.06	3	41%	5	512
Implant breakage	0.01	0.00–0.03	3	32%	4	529
*Subgroup OPRA implants*						
Overall	0.55	0.47–0.64	71	0%	2	128
Abutment breakage	0.67	0.31–0.90	547	98%	5	726
Any mechanical failure	0.68	0.34–0.90	552	98%	5	726
Any infection	0.30	0.13–0.55	174	94%	5	478
Pain	0.12	0.01–0.60	16	87%	3	180
Stump revision	0.07	0.03–0.16	13	53%	2	177
Bone fracture	0.02	0.01–0.03	10	0%	4	653
Aseptic loosening	0.02	0.01–0.03	8	0%	2	480
Implant breakage	0.01	0.00–0.03	1	0%	4	347

Complications as a proportion of all adverse events

^*^Breakage of intramedullary stem, **Only studies with ILP/OPL implants reported on hypergranulation. CI: Confidence interval, ILP: Integral-leg-prosthesis (Orthodynamics), OPL: Osseointegration prosthetic limb (Permedica), OPRA: Osseointegrated prostheses for the rehabilitation of amputees (Integrum)

### Infection

Table 3 summarises infection data. Of 14 studies suitable for analysis [14, 23–25, 27–30, 32, 33, 35–38], eight [14, 24, 27, 30, 32, 33, 35, 36] were suitable for grading under the OGAAP scale.

**Table 3:** Infections, graded, any follow-up

Infection Grade	Proportion	95% CI	Events	I^2^	Included studies (n)	Sample size (n)
Grade I	0.72	0.57–0.83	177	72%	7	260
Grade II	0.23	0.12–0.39	73	77%	7	260
Grade III	0.06	0.02–0.15	14	53%	8	416
Grade IV	0.03	0.01–0.08	4	32%	8	416
*Subgroup ILP/OPL implants*						
Grade I	0.68	0.35–0.90	78	79%	2	134
Grade II	0.36	0.15–0.64	55	61%	3	135
Grade III	0.04	0.02–0.10	8	34%	4	291
Grade IV	0.02	0.01–0.07	2	37%	4	291
*Subgroup OPRA implants*						
Grade I	0.77	0.58–0.89	95	73%	2	121
Grade II	0.15	0.10–0.23	18	0%	2	121
Grade III	0.04	0.00–0.56	6	82%	2	121
Grade IV	0.02	0.00–0.18	2	51%	2	121

CI: Confidence interval, ILP: Integral-Leg-Prosthesis (Orthodynamics), OPL: Osseointegration Prosthetic Limb (Permedica), OPRA: Osseointegrated Prostheses for the Rehabilitation of Amputees (Integrum)

At short-term follow-up, 23% (n = 28) of all adverse events were any type of infection (95% CI: 0.018–0.83, I^2^ = 93%). Mid-term follow-up demonstrated 35% (n = 122) (95% CI: 0.28–0.43, I^2^ = 44%), and long-term 47% (n = 294) (95% CI: 0.23–0.73, I^2^ = 97%) (Table 4**, **SI: Figure S3).

**Table 4:** Infections, any grade, specified follow-up

Follow-up	Proportion	95% CI	Events	I^2^	Included studies (n)	Sample size (n)
12 months	0.23	0.02–0.83	28	93%	2	80
13–59 months	0.35	0.28–0.43	122	44%	6	360
≥ 60 months	0.47	0.23–0.73	294	97%	4	543

CI: Confidence interval

Virtually all graded infections were of the soft tissue. Grade I infections made up the largest portion at 72% (n = 177) (95% CI: 0.57–0.83, I^2^ = 72%), followed by grade II at 23% (n = 73) (95% CI: 0.12–0.39, I^2^ = 77%). Bone infection (grade III) and septic loosening (grade IV) were less common at 6% (n = 14) (95% CI: 0.02–0.15, I^2^ = 53%) and 3% (n = 4) (95% CI: 0.01–0.08, I^2^ = 32%), respectively. After performing a leave-one-out sensitivity analysis, Reetz et al., 2020 was excluded from grade I and II infection analysis for significantly influencing the result (SI: Fig. S3).

Subgroup analysis demonstrated ILP/OPL implants had a total infection rate of 51% (n = 295) (95% CI: 0.38–0.63, I^2^ = 90%). OPRA implants had a total infection rate of 30% (n = 143) (95% CI: 0.13–0.55, I^2^ = 94%).

### Mechanical complications

**Fig. 2:**
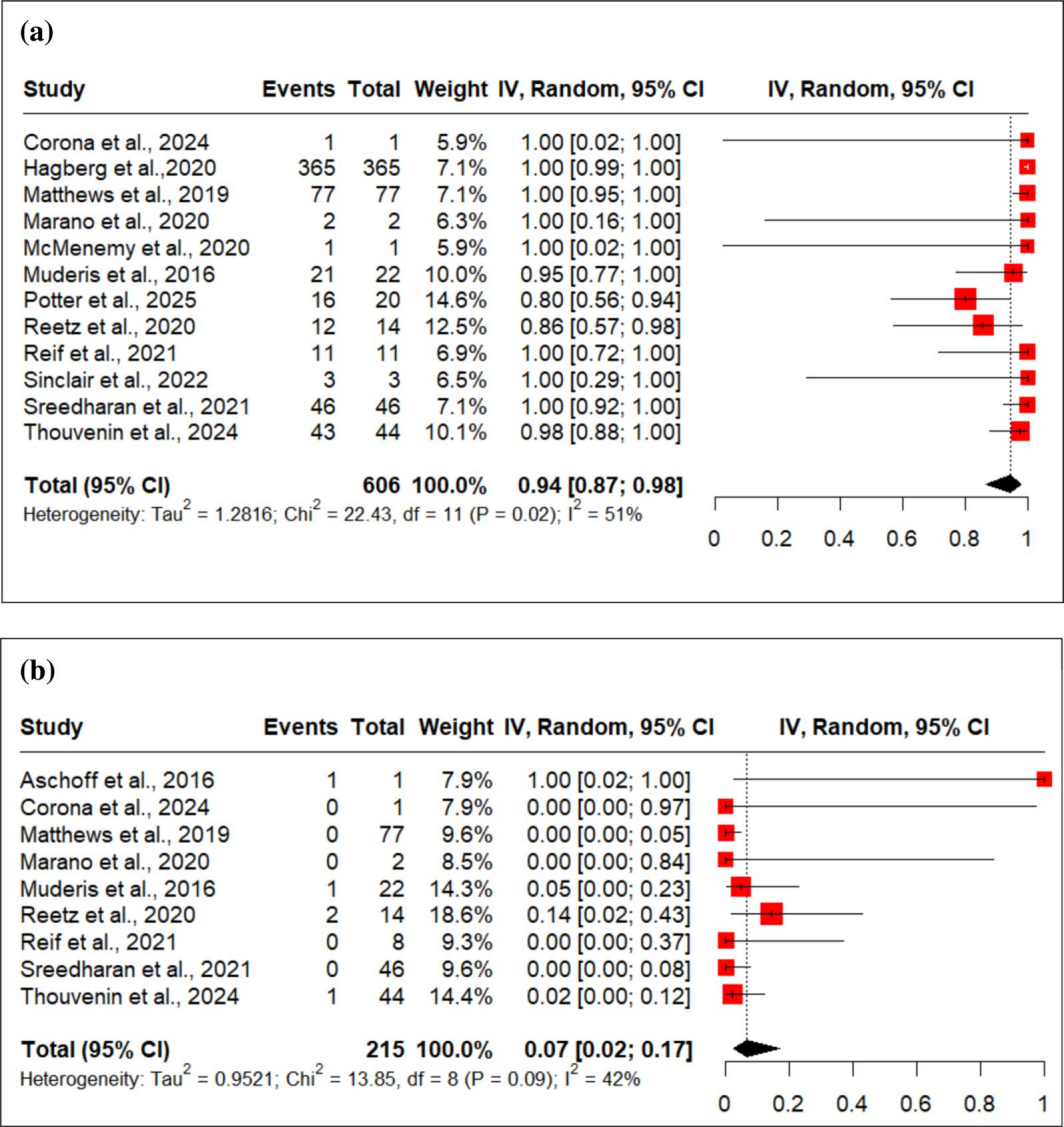
Standalone mechanical complications. **a** Abutment breakage **b** Implant breakage

Standalone analysis of only mechanical adverse events (Fig. 2) demonstrated abutment/dual-cone adaptor breakages constituted nearly all mechanical failures: 94% (n = 598) (95% CI: 0.87–0.98, I^2^ = 51%).

**Fig. 3:**
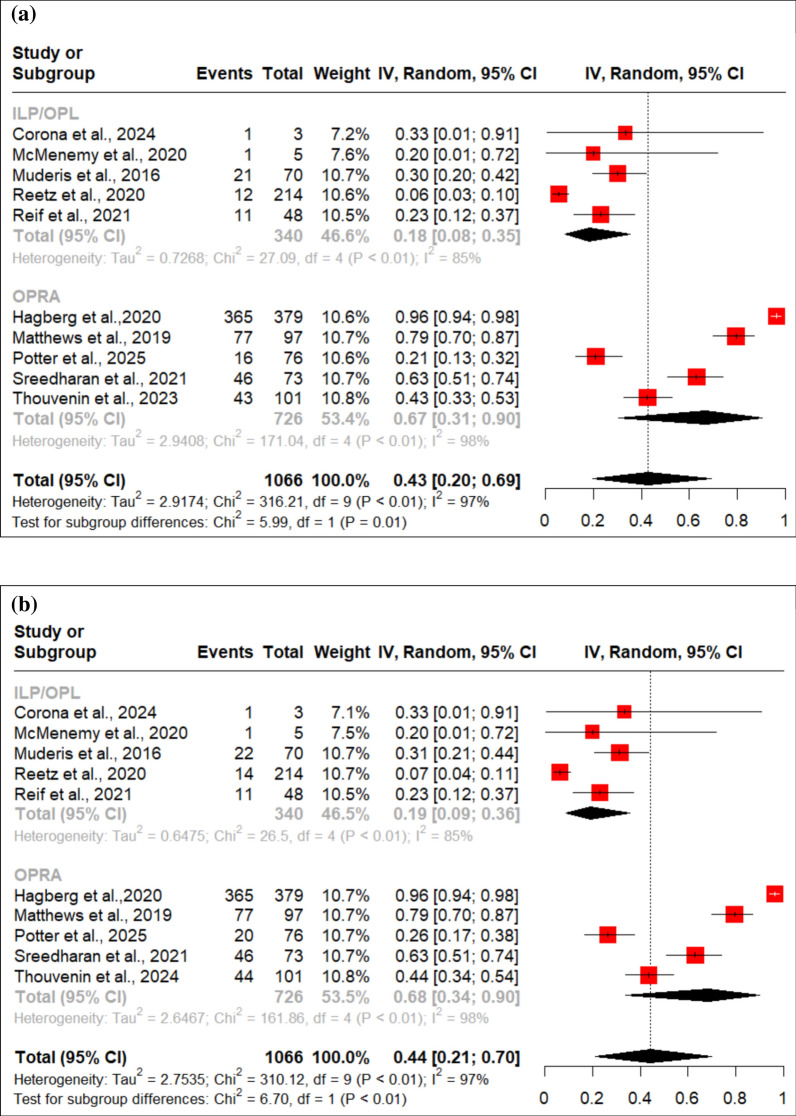
Abutment/dual-cone breakage and overall mechanical failure as a proportion of all adverse events—subgroup analysis. **a** Abutment/dual-cone breakage **b** Overall mechanical failure ILP: Integral-Leg-Prosthesis, OPL: Osseointegrated Prosthetic Limb, OPRA: Osseointegrated Prostheses for the Rehabilitation of Amputees

Across 5 studies [15, 25, 29, 30, 37], subgroup analysis demonstrated OPRA implants to have an abutment breakage rate of 67% (n = 547) (95% CI: 0.31–0.90, I^2^ = 97%) compared to 5 studies [14, 18, 27, 28, 36] reporting on the DCA of the ILP/OPL implants at 18% (n = 46) (95% CI: 0.08–0.35, I^2^ = 85%) (Fig. 3). ORPA implants had an overall mechanical failure of 68% (n = 552) (95% CI: 0.34–0.90, I^2^ = 98%), whilst for ILP/OPL implants it was 19% (n = 49) (95% CI: 0.09–0.36, I^2^ = 85%). Subgroup analyses for pain, bone fracture, aseptic loosening and implant breakage are summarised in Table 2**.** Subgroup analysis of mechanical adverse events is summarised in Table 5**.**

**Table 5:** Mechanical complications

Complication	Proportion	95% CI	Events	I^2^	Included studies (n)	Sample size (n)
Abutment/dual-cone breakage	0.94	0.87–0.98	598	51%	12	606
Implant breakage	0.07	0.02–0.17	5	42%	9	215
Subgroup ILP/OPL						
Abutment/dual-cone breakage	0.89	0.76–0.96	46	0%	5	49
Implant breakage	0.10	0.04–0.24	3	0%	4	45
Subgroup OPRA						
Abutment/dual-cone breakage	0.98	0.89–1.00	547	78%	5	552
Implant breakage	0.01	0.00–0.05	1	0%	3	167

Implant and abutment/dual-cone breakage as a proportion of all recorded mechanical complications

CI: Confidence interval, ILP: Integral-leg-prosthesis (Orthodynamics), OPL: Osseointegration prosthetic limb (Permedica), OPRA: Osseointegrated prostheses for the rehabilitation of amputees (Integrum)

### Implant survival

**Fig. 4:**
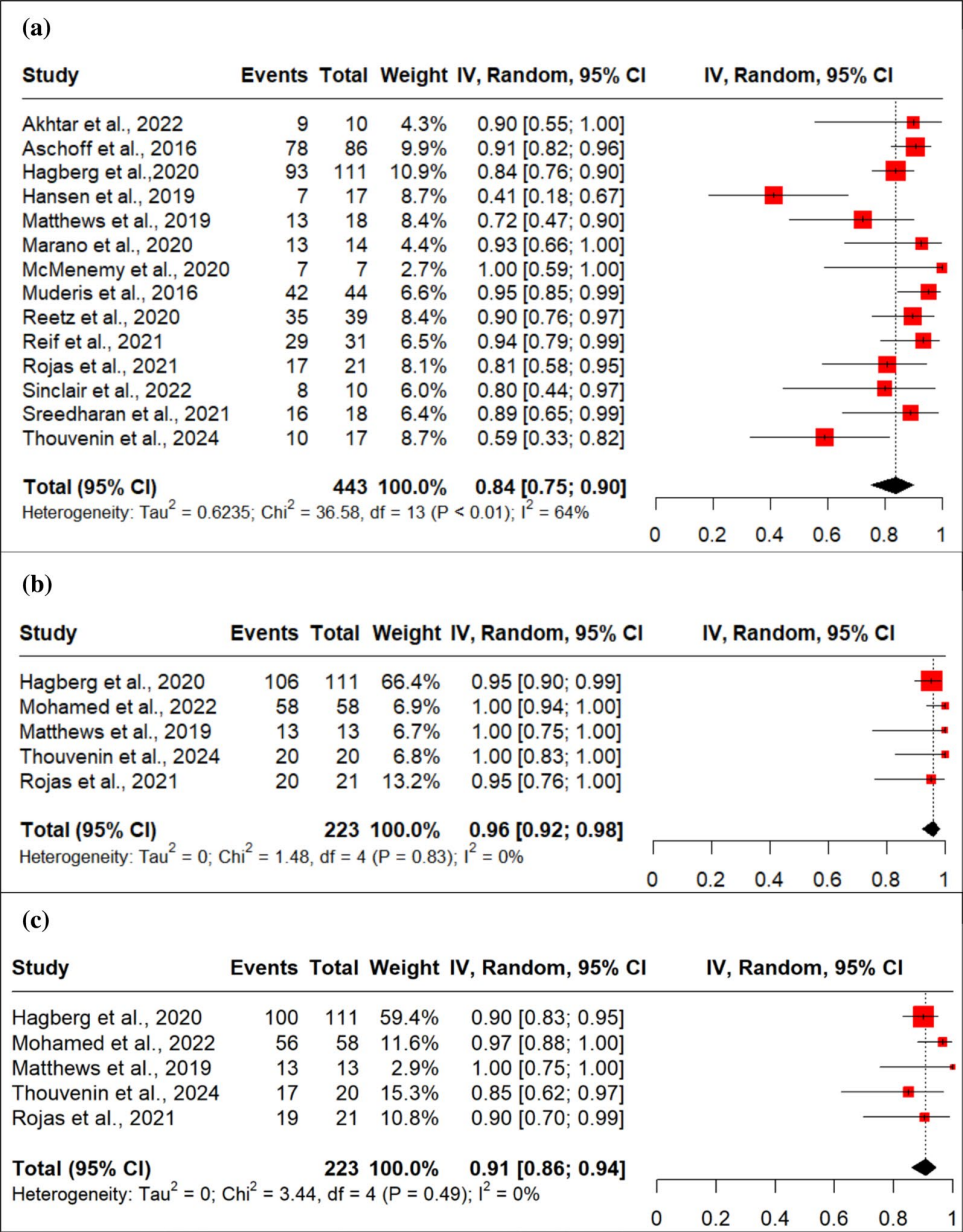

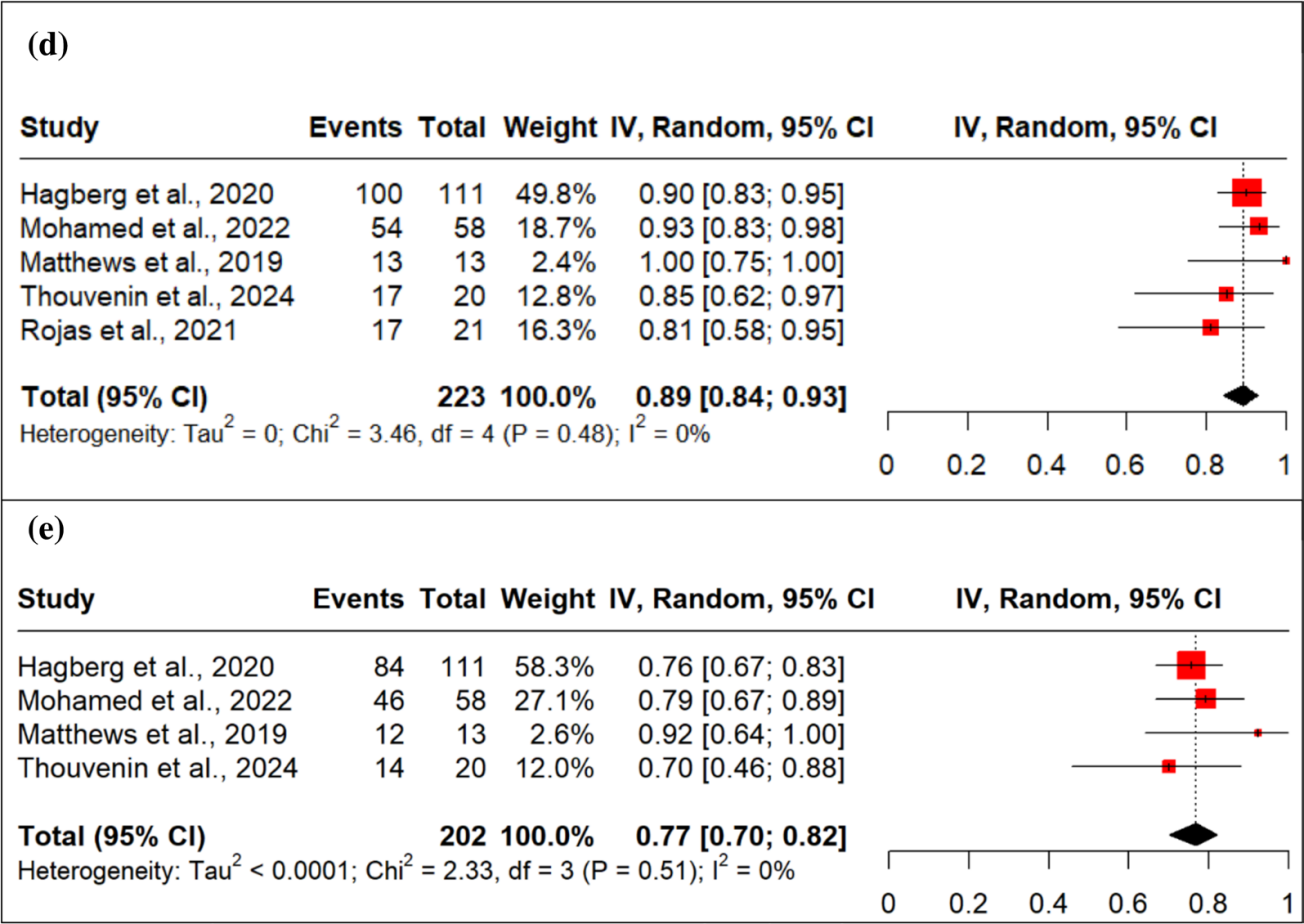
Survival. **a** Survival across all studies, any follow-up **b** 1 year survival **c** 3 year survival **d** 5 year survival **e** 10 year survival

Implant survival is summarised in Fig. 4. Fourteen studies reported on revision and/or failure of implants [14–16, 18, 22, 24, 25, 27–31, 35, 38]. Across these studies is a revision-free survival rate of 84% (95% CI: 0.75–0.90, I^2^ = 64%). Kaplan–Meier curves for implant failure or revision were reported by five studies [15, 25, 26, 30, 31]. Estimated survival at 1 year is 96% (95% CI: 0.92–0.98, I^2^ = 0%). At 3 years, survival is 91% (95% CI: 0.86–0.94, I^2^ = 0%). At 5 years, survival is 89% (95% CI: 0.84–0.93, I^2^ = 0%). At 10 years, survival falls to 77% (95% CI: 0.70–0.82, I^2^ = 0%).

Reasons for explantation/revision included infection (deep and superficial), bone fracture, pain, aseptic loosening and mechanical issues. Eight studies reported on management of all removed implants [16, 24, 26–28, 33, 38]. Most commonly, all hardware was removed (77%, n = 44). Other approaches included removing only the abutment/DCA and closing the stump around the intramedullary component. Subsequent successful reimplantation for all cases of total removal was reported by three studies [24, 26, 38]. Reported in three studies, few explantations were partial removals (removal of abutment/DCA only) [16, 26, 27]. Nine other studies reported at least one explantation/revision but did not comprehensively report management [14, 17, 22, 23, 25, 29, 30, 34, 35].

## Discussion

Augmentation for lower-limb amputees remains a continually developing field in which BAPs may be a viable alternative for SSP users. However, concerns regarding infection and implant breakage hinder widespread clinical implementation. We identified an overall complication rate of 65%. When analysing all complication events, 36% are expected to be due to skin and soft tissue infection. The remaining majority are from breakage of the abutment/DCA, followed by revision of the stump. Implant survival remains high with ongoing use, with up to 77% unrevised within a 5–10-year period. To the authors’ knowledge, this is the first meta-analysis comprehensively demonstrating the safety of BAPs in lower-limb amputees.

Total hip arthroplasty (THA) has previously been employed as a frame of reference by Atallah et al. to discuss BAP complications. Although an imperfect comparison, it remains useful to benchmark infection and survival until further dedicated BAP literature emerges. A database meta-analysis found approximately 0.34–2.45% of patients developed periprosthetic joint infection following THA [39]. In contrast, the present study found an infection rate of 36% for BAPs. At face value, infection is a far more prominent issue for BAPs than of more established orthopaedic interventions. Despite this, risk factors for BAP-related infections are not well elucidated, prohibiting any definitive statement on infection. For example, it is not definitively known if transtibial or transfemoral implantation influences BAP-related infection [40]. Impact of surgical techniques and implant design has seen some exploration, however. A systematic review has indicated single-stage implantation is likely superior to a two-stage approach, but was beyond the scope of the present study [41]. Juhnke and colleagues established that some designs are better than others, reporting no post-implantation infections after updating the ILP design [23]. Yet, follow-up for this group is short (~ 4 y) compared to the Swedish OPRA cohort (15 y)[34]. Time is likely relevant when considering infections as Hagberg et al. 2023 reported a higher prevalence of superficial infections during the first five years with decreasing rates in the second five-year period [34]. To compliment this, analysis of osteomyelitis in the Swedish cohort identified time since implantation as a risk factor for infection, and evidence for a relationship between first abutment change and subsequent infection [19]. More research is needed to explore these notions as our findings were similar across follow-up periods.

It remains to be seen if the press-fit ILP/OPL or screw-fit OPRA design suffers fewer infections. In our analysis, overall rates of infection (ILP/OPL 51% against OPRA 30%) are probably resultant of comprehensive reporting of mechanical failures, which are apparently more common over longer follow-up–both present in OPRA studies [15, 30, 34]. Graded infection analysis indicated severity is broadly comparable between the two groups, i.e. usually limited to soft tissue. This is positive, as multiple studies report grade I and II infections are generally manageable with oral antibiotics. Nonetheless, some cases still require parenteral antibiotics and/or debridement [14, 18, 24, 25, 27, 28, 30, 33, 42, 43]. Ultimately the findings of this meta-analysis highlight the need for a robust two-arm study with both implants.

Mechanical complications also appear common, but not totally detrimental. If safety mechanisms fail, the abutment or DCA can sacrificially safeguard components further upstream. Under very high load, a properly balanced abutment/DCA should break, necessitating replacement only of itself, a naturally less invasive procedure than complete revision. The current meta-analysis reflects this, finding both implant breakages (1%) and bone fractures (4%) uncommon. This is concordant with previous literature stating periprosthetic fractures are rare (6.3%), usually occurring after falls [44]. As such, our findings demonstrate stakeholders need not worry excessively about high-risk BAP implant breakage. During biomechanical analysis, Mirulla et al. found that compared to OPRA implants, press-fit OPL implants generate larger high-stress areas in the distal femur under critical loads [45]. This is likely at least partially responsible for the higher rates of bone fracture we observed in ILP/OPL implants (5%). Nearly all mechanical failures analysed were attributed to the abutment or DCA, indicating relative safety in current designs. Regardless, this “sacrificial” mechanism has pitfalls. Younger and/or particularly active patients are penalised with higher rates of abutment failure, a phenomenon noted by multiple authors [15, 29, 33]. Additional data regarding abutment and implant strength, patient activity levels and body mass index (BMI) are required.

The survival rate analysed across all current studies is promising. To the best of the authors’ knowledge, this is the first time estimated BAP survival has been reported across discrete follow-up periods in a review. Hence, there is no established reference literature for concordance of results, so THA is again our reference frame. A scoping review of THA patients aged ≤ 55 reported survival at 5 years ranging from 90 to 100% and at 10 years 62–98% [46]. A smaller review on the same age group reported 95–100% survival at 5 years and 78.1–100% at 10 years [47]. Our meta-analysis indicated implant survival at 5 years is nearly 90% but falls appreciably at 10 years to 77%. These results are positive, demonstrating the longevity of BAPs is currently in-line with the mid- to lower-end of THA survival estimates.

Naturally, numerous variables influence survival rates. A relationship between recency of surgery and THA survival has been identified, i.e. during the same follow-up period, more-recent surgeries demonstrate better survival [46, 48]. This is likely also true for BAPs, as Matthews et al. noted improved BAP survival following formalisation of the OPRA protocol in 1999. Juhnke and Aschoff subsequently reported no infection-related revisions after redesigning the ILP in 2009 [17, 25]. The implication being that BAP survival is a still-evolving metric and likely to improve in studies with newer cohorts and more experienced clinicians. Second, Evans et al. reported a strong correlation between age, sex and implant selection and THA survival [49]. In our survival analysis, patients were of a reasonably similar age, mostly males with traumatic amputation and treated with OPRA implants. It is still uncertain if sex, age and/or amputation aetiology have an appreciable influence on BAP survival. The Swedish team identified an association with early infection and later implant removal, whilst the Dutch similarly reported a high number of infectious events as a risk factor for failure [26, 34]. As with infection, implant design is an important variable for BAP survival. In an ILP implant cohort, Mohammed et al. observed an association between small intramedullary stem diameter (15–16 mm) and implant breakage [26]. Despite low rates of implant breakage and acceptable survival, BAP revision is of concern as ideal outcomes appear to require highly-specialised tools, and septic implants may necessitate extensive debridement and compaction grafting [50]. Further studies focused on identifying risk-factors for BAP failure across long-term cohorts are needed.

There is a modest base of research investigating BAPs for lower-limb amputees, but quality is highly variable. A special interest group (SIG) for BAPs under the International Society for Prosthetics and Orthotics (ISPO) is developing the Bone-Anchored Lower Limbs Core Outcome Set (BALLCOS) [51]. Consensus-driven standardisation of outcomes is essential, and all future lower-limb BAP research should abide by finalised BALLCOS guidelines. A requirement within these guidelines for reporting both number of patients affected and the number of events occurring for a given complication is vital for future elaboration on the findings here.

The group also lists the exploration of a need for a global data management system to facilitate joint publications as one objective [52]. An individual patient data (IPD) meta-analysis would be more robust than the present study, but data for this are lacking. Hence, the authors see establishment of a central IPD repository as a worthwhile initiative.

Additionally, clarity in reporting periods and cohort overlap should be prioritised in the future papers. Alongside the BALLCOS, recommended milestone reporting periods for prospective studies, i.e. 1 year, 3 years, 5 years, 10 years, should be agreed as our findings support Delphi-established outcomes of clinical interest within these achievable time frames [53]. Furthermore, present literature commonly mixes any number of different cohorts, often not clearly reported and subsequently obfuscating the true data to anyone except the original authors. Exclusion of multiple eligible studies in the present meta-analysis was the direct result of this practice [26, 42, 43, 54]. Authors should endeavour to report a complete BALLCOS-compliant paper on a given cohort before publishing literature with more focused research aims or partial cohorts.

## Limitations

There was sufficient evidence to address the main outcomes of interest. But as with all meta-analyses, limitations are present. This review was limited by the small sample size of included studies—the largest being n = 111. Some publication bias exists due to the relatively small pool of researchers publishing in this area (SI: Figure S9). Subgroup analysis for the most common implant designs (OPRA and ILP/OPL) was possible; however, ILP and OPL implants were analysed as one subgroup. Both are press-fit designs of similar lineage, and several studies used the ILP and OPL implants in parallel without distinction in reported data. Subgroup analysis for outcomes in transfemoral and transtibial amputees was not performed due to the small number of transtibial amputees (n = 61) and lack of discrete reporting of outcomes by amputation level in some studies.

Irrespective of follow-up or population, many included studies suffer from bias from failure to identify or control confounding factors and selective reporting. Selective reporting and failure to collect or report important data necessitated a limited approach in some areas.

Several studies have limited (< 3 year) follow-up which is insufficient to observe all possible events/outcomes. Hence, proportions of infections and adverse events are likely underreported, with a subsequent overestimation in our analysed proportions. Only infections and survival were pooled at discrete follow-up points. Outpatient infection management and subsequent failure to record in study data are not unusual, and the grading scale we used for infection is unvalidated, possibly skewing data. Management of revision and implant removal is not comprehensively reported, nor was it a focus in this review. Hence, our findings regarding the safety profile of BAPs can only be applied until revision is required.

Heterogeneity is high for several analyses, primarily due to shortcomings in source literature. There is significant variance in follow-up time, and how complications were defined and recorded. Most studies lacked discrete follow-up periods for complications and most authors did not respond to data requests. However, the authors believe the heterogeneity and other limitations are permissible, given the results generally reflect the existing consensus and serve to reinforce, not challenge.

Implant design, surgical implantation protocol and rehabilitation protocol have been flagged by others as consequential in the outcomes of lower-limb BAPs. In-depth exploration of these areas was beyond the present scope.

## Conclusion

Low-grade (grade I) skin infections and breakages of the abutment/DCA are the most common complications which both patient and clinician should account for in BAPs. Revision-free survival rates are consistently high up to five years and remain acceptable at ten years when compared to more established orthopaedic procedures. Differences do exist for rates of mechanical complications between the most common implant designs. Clinically, BAPs appear as reasonably safe alternatives for lower-limb amputees but carry appreciable risks and limitations. Namely patients should be aware of the likelihood of requiring treatment for skin infections and the prospect of additional procedures to deal with failed abutments or dual-cone adaptors.

## Supplementary Information

Below is the link to the electronic supplementary material.

Supplementary file1 (DOCX 4102 KB)

## Data Availability

Upon request the dataset used for this meta-analysis will be made available by the authors.

## Abbreviations

BAP: Bone-anchored prosthesis
BMI: Body mass index
BALLCOS: Bone-anchored lower limbs core outcome set
CI: Confidence interval
OGAAP: Osseointegration group of Australia accelerated protocol
ROBINS-I: Risk of bias in non-randomised studies of interventions
SSPs: Suspended socket prostheses
REML: Restricted maximum likelihood
ILP: Integral-leg-prosthesis
OPL: Osseointegration prosthetic limb
OPRA: Osseointegrated prostheses for the rehabilitation of amputees
THA: Total hip arthroplasty
TFA: Transfemoral amputation
TTA: Transtibial amputation
DCA: Dual-cone adaptor
