# Supplementary material for: Meta-analytic evaluation of complications and longevity of bone-anchored prostheses in lower extremity amputees: does design matter?
Source: Musculoskelet Surg. 2026 Jan 14;110(3):287–301. doi: 10.1007/s12306-026-00946-9 (PMC13473266; doi:10.1007/s12306-026-00946-9)
Supplement: Supplementary file 1 [file 12306_2026_946_MOESM1_ESM.docx]

**Supplementary Information**

**Item 1: Complete search strategy**

Searches

PubMed

**((Bone-anchored Prosthesis) OR ((("bone anchored") OR (osseointegration)) AND (("artificial limb" OR "artificial limbs" OR "prosthetic limb" OR "limb prosthesis") OR (artificial limb))))**

**AND**

**(((lower extremity amputation) OR (lower limb amputation)) OR (transfemoral amputation))**

**Results in 156 hits**

**Update 27-2-24 to 6-2-25	31 hits**

Embase

Session Results

.......................................................

No. Query Results	Results Date

#16. #10 AND #13 AND [01-01-2000]/sd NOT	137 31 Aug 2025

[07-02-2025]/sd

#15. #10 AND #13 AND [27-02-2024]/sd NOT	21 31 Aug 2025

[07-02-2025]/sd

#14. #10 AND #13	159 31 Aug 2025

#13. #11 OR #12	20,276 31 Aug 2025

#12. 'amputation, leg' OR 'lower extremity amputation'	13,996 31 Aug 2025

OR 'lower limb amputation' OR 'leg amputation' OR

'transfemoral amputation'

#11. 'leg amputation'/exp	17,685 31 Aug 2025

#10. #1 OR #9	421 31 Aug 2025

#9. #5 AND #8	421 31 Aug 2025

#8. #6 OR #7	11,951 31 Aug 2025

#7. 'artificial extremity' OR 'artificial limb' OR	7,068 31 Aug 2025

'artificial limbs' OR 'bone-anchored prosthesis'

OR 'external limb prostheses' OR 'external limb

prosthesis' OR 'limb prosthesis device' OR

'prosthesis, limb' OR 'prosthetic limb' OR 'limb

prosthesis'

#6. 'limb prosthesis'/exp	11,110 31 Aug 2025

#5. #2 OR #3 OR #4	17,816 31 Aug 2025

#4. 'osseo-integration' OR 'osseous integration' OR	15,872 31 Aug 2025

'osseointegration'

#3. 'osseointegration'/exp	7,928 31 Aug 2025

#2. 'bone-anchored'	2,302 31 Aug 2025

#1. 'bone-anchored prosthesis'	107 31 Aug 2025

.......................................................

Search Name:

Date Run:	01/09/2025 02:31:23

Comment:

ID	Search	Hits

#1	MeSH descriptor: [Bone-Anchored Prosthesis] explode all trees	12

#2	("bone anchored prosthesis"):ti,ab,kw (Word variations have been searched)	12

#3	#1 OR #2	12

#4	("bone anchored"):ti,ab,kw (Word variations have been searched)	151

#5	MeSH descriptor: [Osseointegration] explode all trees	458

#6	(osseointegration):ti,ab,kw (Word variations have been searched)	1153

#7	#4 OR #5 OR #6	1294

#8	MeSH descriptor: [Artificial Limbs] explode all trees	252

#9	(prosthetic limb):ti,ab,kw (Word variations have been searched)	526

#10	(artificial limb):ti,ab,kw (Word variations have been searched)	654

#11	(limb prosthesis):ti,ab,kw (Word variations have been searched)	1028

#12	{OR #8-#11}	1582

#13	#7 AND #12	5

#14	#13 OR #3	17

#15	MeSH descriptor: [Lower Extremity] explode all trees	10378

#16	((Extremity, Lower OR Lower Extremities OR Limbs, Lower OR Membrum inferius OR Limb, Lower OR Lower Limbs OR Lower Limb OR Extremities, Lower)):ti,ab,kw (Word variations have been searched)	32232

#17	#15 OR #16	38768

#18	MeSH descriptor: [Amputation, Surgical] explode all trees	709

#19	(amputation):ti,ab,kw (Word variations have been searched)	4430

#20	#18 OR #19	4431

#21	#17 AND #20	1841

#22	#14 AND #21	1

Scopus

Scopus

((TITLE-ABS-KEY("Bone-anchored Prosthesis")) OR ((TITLE-ABS-KEY("Bone anchored" OR osseointegration)) AND (TITLE-ABS-KEY("artificial limb*" OR "prosthetic limb*" OR "limb prosthes*" OR "'artificial extremity")))) AND ((TITLE-ABS-KEY("lower limb" OR "lower extremity" OR transfemoral OR leg)) AND (TITLE-ABS-KEY(amputat*)))

27-2-2024-7-2-2025 **45**

\# Web of Science Search Strategy (v0.1)

\# Database: Web of Science Core Collection

\# Entitlements:

- WOS.IC: 1993 to 2025

- WOS.CCR: 1985 to 2025

- WOS.SCI: 1900 to 2025

- WOS.AHCI: 1975 to 2025

- WOS.BHCI: 2005 to 2025

- WOS.BSCI: 2005 to 2025

- WOS.ESCI: 2005 to 2025

- WOS.ISTP: 1990 to 2025

- WOS.SSCI: 1900 to 2025

- WOS.ISSHP: 1990 to 2025

\# Searches:

1: TS=("Bone-anchored Prosthesis")	Date Run: Mon Sep 01 2025 10:22:14 GMT+1000 (Australian Eastern Standard Time)	Results: 93

2: TS=(("Bone-anchored" OR osseointegration) AND ("artificial limb*" OR "prosthetic limb*" OR "limb prosthes*" OR "'artificial extremity"))	Date Run: Mon Sep 01 2025 10:22:46 GMT+1000 (Australian Eastern Standard Time)	Results: 185

3: #2 OR #1	Date Run: Mon Sep 01 2025 10:23:07 GMT+1000 (Australian Eastern Standard Time)	Results: 242

4: TS=(("lower limb" OR "lower extremity" OR transfemoral OR leg) AND amputat*)	Date Run: Mon Sep 01 2025 10:23:40 GMT+1000 (Australian Eastern Standard Time)	Results: 18224

5: #3 AND #4	Date Run: Mon Sep 01 2025 10:23:58 GMT+1000 (Australian Eastern Standard Time)	Results: 154

6: #3 AND #4	Timespan: 2024-02-27 to 2025-02-06	Date Run: Mon Sep 01 2025 10:26:21 GMT+1000 (Australian Eastern Standard Time)	Results: 18

CiNAHL

| **#** | **Query** | **Limiters/Expanders** | **Last Run Via** | **Results** |
| --- | --- | --- | --- | --- |
| S9 | S7 AND S8 | Expanders - Apply equivalent subjects Search modes - Boolean/Phrase | Interface - EBSCOhost Research Databases Search Screen - Advanced Search Database - CINAHL Complete | 77  Limited to 27-02-24-6-02-25 13 |
| S8 | lower extremity amputation OR lower limb amputation OR transfemoral amputation OR leg amputation | Expanders - Apply equivalent subjects Search modes - Boolean/Phrase | Interface - EBSCOhost Research Databases Search Screen - Advanced Search Database - CINAHL Complete | 6,758 |
| S7 | S1 OR S6 | Expanders - Apply equivalent subjects Search modes - Boolean/Phrase | Interface - EBSCOhost Research Databases Search Screen - Advanced Search Database - CINAHL Complete | 482 |
| S6 | S2 AND S5 | Expanders - Apply equivalent subjects Search modes - Boolean/Phrase | Interface - EBSCOhost Research Databases Search Screen - Advanced Search Database - CINAHL Complete | 408 |
| S5 | S3 OR S4 | Expanders - Apply equivalent subjects Search modes - Boolean/Phrase | Interface - EBSCOhost Research Databases Search Screen - Advanced Search Database - CINAHL Complete | 18,246 |
| S4 | artificial limb* OR prosthetic limb* OR limb prosthesis | Expanders - Apply equivalent subjects Search modes - Boolean/Phrase | Interface - EBSCOhost Research Databases Search Screen - Advanced Search Database - CINAHL Complete | 18,246 |
| S3 | (MM "Limb Prosthesis") | Expanders - Apply equivalent subjects Search modes - Boolean/Phrase | Interface - EBSCOhost Research Databases Search Screen - Advanced Search Database - CINAHL Complete | 2,267 |
| S2 | "bone anchored" OR osseointegration | Expanders - Apply equivalent subjects Search modes - Boolean/Phrase | Interface - EBSCOhost Research Databases Search Screen - Advanced Search Database - CINAHL Complete | 2,922 |
| S1 | Bone-anchored Prosthesis | Expanders - Apply equivalent subjects Search modes - Boolean/Phrase | Interface - EBSCOhost Research Databases Search Screen - Basic Search Database - CINAHL Complete | 217 |

**Figure 1: ROBINS-I Risk of bias assessment**

**Figure 2: Contour-enhanced funnel plots**

A: Grade I infections

B: Grade II infections

**Figure 3: Leave-one-out sensitivity analysis for graded infections I-II**
